# Patient factors associated with new prescribing of potentially inappropriate medications in multimorbid US older adults using multiple medications

**DOI:** 10.1186/s12877-021-02089-x

**Published:** 2021-03-06

**Authors:** Katharina Tabea Jungo, Sven Streit, Julie C. Lauffenburger

**Affiliations:** 1 Institute of Primary Health Care (BIHAM), University of Bern, Bern, Switzerland; 2 Department of Epidemiology, Harvard T.H. Chan School of Public Health, Boston, MA USA; 3 Division of Pharmacoepidemiology and Pharmacoeconomics, Department of Medicine, Brigham and Women’s Hospital and Harvard Medical School, Boston, MA USA; 4 Graduate School for Health Sciences, University of Bern, Bern, Switzerland; 5 Center for Healthcare Delivery Sciences, Department of Medicine, Brigham and Women’s Hospital, Boston, MA USA

**Keywords:** Multimorbidity, Polypharmacy, Potentially inappropriate prescribing

## Abstract

**Background:**

The use of potentially inappropriate medications (PIMs) is common in older adults and is associated with potential negative consequences, such as falls and cognitive decline. Our objective was to investigate measurable patient factors associated with new outpatient prescribing of potentially inappropriate medications in older multimorbid adults already using multiple medications.

**Methods:**

In this retrospective US cohort study, we used linked Medicare pharmacy and medical claims and electronic health record data from a large healthcare system in Massachusetts between 2007 and 2014. We identified patients aged ≥65 years with an office visit who had not been prescribed or used a PIM in the prior 180 days. PIMs were defined using 2019 Beers criteria of the American Geriatrics Society. To specifically evaluate factors in patients with polypharmacy and multimorbidity, we selected those who filled medications for ≥90 days (i.e., chronic use) from ≥5 pharmaceutical classes in the prior 180 days and had ≥2 chronic conditions. Multivariable Cox regression analysis was used to estimate the association between baseline demographic and clinical characteristics on the probability of being prescribed a PIM in the 90-day follow-up period.

**Results:**

In total, we identified 17,912 patients aged ≥65 years with multimorbidity and polypharmacy who were naïve to a PIM in the prior 180 days. Of those, 10,497 (58.6%) were female, and mean age was 78 (SD = 7.5). On average, patients had 5.1 (SD = 2.3) chronic conditions and previously filled 6.1 (SD = 1.4) chronic medications. In total, 447 patients (2.5%) were prescribed a PIM during the 90-day follow-up. Male sex (adjusted hazard ratio (HR) = 1.29; 95%CI: 1.06–1.57), age (≥85 years: HR = 0.75, 95%CI: 0.56–0.99, 75–84 years: HR = 0.87, 95%CI: 0.71–1.07; reference: 65–74 years), ambulatory visits (18–29 visits: HR = 1.42, 95%CI: 1.06–1.92; ≥30 visits: HR = 2.12, 95%CI: 1.53–2.95; reference: ≤9 visits), number of prescribing orders (HR = 1.02, 95%CI: 1.01–1.02 per 1-unit increase), and heart failure (HR = 1.38, 95%CI: 1.07–1.78) were independently associated with being newly prescribed a PIM.

**Conclusion:**

Several demographic and clinical characteristics, including factors suggesting lack of care coordination and increased clinical complexity, were found to be associated with the new prescribing of potentially inappropriate medications. This knowledge could inform the design of interventions and policies to optimize pharmacotherapy for these patients.

**Supplementary Information:**

The online version contains supplementary material available at 10.1186/s12877-021-02089-x.

## Introduction

The prevalence of older adults is growing in the United States and many countries globally, in large part because of increasing life expectancy [1]. At the same time, the prevalence of multimorbidity, commonly defined as having ≥2 chronic conditions [2], is also on the rise [3]. Accordingly, due to the association between multimorbidity and age [4], multimorbidity is becoming increasingly common in older adults. Multimorbidity poses one of the greatest challenges to health systems, because multimorbid patients often have complex healthcare needs and worse health outcomes [5, 6], including higher rates of mortality, disability, lower quality of life, and adverse drug events [7, 8]. Another challenge associated with multimorbidity is the increasing number of medications that patients need to take to manage their conditions.

Multimorbid patients often have polypharmacy, i.e., the concurrent use of ≥5 medications [9]. For instance, 39% of community-dwelling US older adults have polypharmacy [10]. Polypharmacy increases the risk of using potentially inappropriate medications (PIMs) [8–10]. PIMs are drugs for which the risk of potential adverse events is greater than the clinical benefits, particularly when there are safer or more effective alternatives that are recommended to be used in older adults [11]. In specific, PIMs are associated with increased risk of adverse drug events, falls, and cognitive impairment [12–15] as well as greater use of healthcare services (e.g., hospitalizations or emergency department visits) and healthcare costs [16–19].

Contributors to the prescribing of PIMs are multifaceted [20]. For example, provider and health-system factors leading to prescribing of PIMs are thought to include lack of communication between different prescribers, providers’ lack of knowledge in geriatric medicine and pharmacology, and insufficient time allocated to prescribing. Previous research on patient factors associated with the prescribing of potentially inappropriate medications for older adults have focused on broad populations of community-dwelling older adults or patients with selected chronic conditions [21–23]. Unfortunately, even though PIM use is high among multimorbid older adults using multiple medications, little is known about the patient factors associated with the new prescribing of potentially inappropriate medications in this population group, despite it being at even greater risk of adverse health outcomes than general older adults.

Therefore, the aim of this study was to explore the factors associated with new prescribing of potentially inappropriate medications in older multimorbid men and women with polypharmacy in the US. Investigating these factors could inform the design of interventions and policies aimed at optimizing pharmacotherapy in this patient group.

## Methods

### Data source

In this retrospective study, we used a dataset containing Medicare claims linked with electronic health records (EHR) of patients enrolled in the Partners Research Patient Data Registry (RPDR) [24]. The Partners Research Patient Data Registry contains EHR data from two tertiary medical centers, three community hospitals, a Rehabilitation center, and a psychiatric hospital that are located in the Boston metropolitan area. The dataset contains data from 569,969 participants from January 1, 2007 through December 31, 2014. Medicare claims include Parts A (inpatient coverage), B (outpatient coverage), and D (drug coverage) containing information on drugs dispensed and start/end dates of insurance coverage [25, 26]. The EHR data contain information on sociodemographic variables, health services use (e.g., ambulatory visits and inpatient care), prescribing records, laboratory tests, and results.

### Patient population

This research uses the same approach to define polypharmacy and multimorbidity as our previous research on the use of potentially inappropriate medications in older multimorbid adults with polypharmacy [27]. The key features of this approach are outlined below.

#### Definition of multimorbidity

We defined the chronic conditions using the Chronic Condition Indicator (CCI) of the Agency for Healthcare Research and Quality (AHRQ). The CCI categorizes ICD-9 diagnosis codes as chronic and not chronic [28]. After extracting the chronic ICD-9 codes, we assigned related codes to ICD-9 code categories. This ensured that we did not misclassify patients with closely-related diagnoses codes (e.g. different types of cancers) as having multiple chronic conditions. Chronic conditions from the CCI related to pregnancy and childbirth were excluded due to their non-relevance in our study population. In total, there were 77 chronic condition categories. To increase the specificity of underlying chronic conditions, ≥2 diagnosis codes on separate days were required for the condition to count as a chronic condition [29]. Because a definition of two or more chronic diseases is commonly used in the literature to define multimorbidity [2, 30, 31], we used this threshold to define patients as multimorbid.

#### Definition of polypharmacy

We used information from the U.S. Food and Drug Administration (FDA) on the classification of medications into different pharmaceutical classes (e.g., anticholinergics) [32] to define polypharmacy. We measured medication use at the class level, as medications with structural similarities, such as statins, are generally considered interchangeable. Medication classes with ≥90 days’ supply were considered as being used chronically [33]. We measured days’ supply from claims conservatively to ascertain long-term polypharmacy. First, we assumed concurrent utilization if there were multiple fills for the same class on the same day, and if the recorded days’ supply differed, the medication with the longest duration was selected. Next, a limited shift of supply (30 days) was used for overlapping utilization in the case of non-concurrent fills. We defined patients as having polypharmacy when they filled medications from ≥5 pharmaceutical classes with ≥90 days’ supply each, in line with commonly used definitions for polypharmacy and previously used approaches for measuring chronic use [9, 34, 35].

#### Cohort definition

**Fig. 1:**
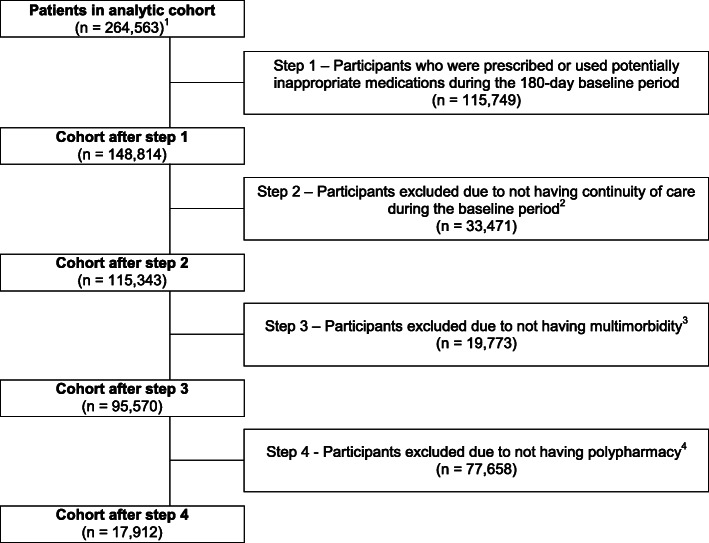
Flow diagram of cohort definition. ^1^cohort entry event = ambulatory visit recorded in RPDR electronic medical records, min. 180 days enrolment in Medicare prior to cohort entry, ≥65 years old, no missing information on sex; ^2^as measured by having min. 1 ambulatory care visit recorded in the RPDR electronic health records during this period; ^3^multimorbidity defined as chronic conditions from ≥2 chronic condition categories; ^4^polypharmacy defined as medications with ≥90 days’ supply each from ≥5 pharmaceutical classes

The cohort was created using the Aetion Evidence Platform (Version: r3.5.20180426_1659), which has previously been validated for a range of studies [25, 36]. An ambulatory visit recorded in the RPDR electronic health records constituted the cohort entry event. From that index ambulatory visit, we excluded patients if they were < 65 years of age, if there was missing information on sex, or if they did not have 180 days Medicare (part D, drug coverage) enrolment prior to the cohort entry to ensure complete data capture. As a result, the effective cohort entry date of patients in our cohort was July 1, 2007 at the earliest. If a patient had multiple possible qualifying ambulatory visits, the patient entered the cohort on the first-occurring qualifying event after exclusions (age, missing sex, and Medicare enrolment) were applied. Patients were only counted once and could not re-enter the cohort at a later stage. Then, we excluded patients who were prescribed or used potentially inappropriate medications during the baseline period, to focus on new PIM prescribing. Next, to ascertain continuity of care and to reduce information bias, we excluded patients who did not have an ambulatory visit during the baseline period of 180 days and for whom there thus was no “data continuity” [37]. Finally, we selected patients with ≥2 chronic conditions and ≥ 5 medication classes with long-term use (i.e., ≥90 days’ supply each), as they were the population of interest. A cohort flow diagram can be found in Fig. 1.

### Outcome measurement

#### Prescribing of potentially inappropriate medications

The identification of potentially inappropriate medications can be done using different implicit (judgment-based) or explicit (criterion-based) lists. The Beers list, published by the American Geriatrics Society (AGS) [38–40], is one example of a criterion-based list. We decided to use the 2019 Beers list, rather than previous versions, to identify potentially inappropriate medications with the aim of informing current medical decision-making [39]. We defined all medications prescribed in the EHR that met any of the drug, duration and dosage requirements described in Table 2 of the 2019 Beers criteria as potentially inappropriate. Certain medications on the Beers list are considered potentially inappropriate only when they are used in presence or absence of a certain diagnosis, when a lab value is below/above a certain value, or when they are used for more than a certain number of days (refer to eTable 1 in the Supplement for details). Using this linked claims and EHR dataset, we were able to capture all the clinical criteria necessary to define PIMs (e.g., diagnoses, lab results).

We measured whether patients in our cohort were newly-prescribed a potentially inappropriate medication in the outpatient setting during a 90-day follow-up period, including the index ambulatory visit (any). For reasons related to continuity of care, we limited our analyses to this 90-day follow-up period. Furthermore, this follow-up period seemed reasonable in our study population with a relatively high health services use (i.e., median number of ambulatory visits during the 180-day baseline period = 17).

#### Covariates

We used peer-reviewed literature to identify patient factors hypothesized to be associated with the prescribing of potentially inappropriate medications and that could also be measured in our EHR-claims dataset [41, 42]. These factors were measured in the 180 days before the index ambulatory visit. The following covariates were included in our models: age, sex, ethnicity, race, number of inpatient stays, emergency department visits, ambulatory visits, non-acute institutional stays, level of polypharmacy (5–9 medications vs ≥10), number of chronic conditions, number of prescribing orders, and selected chronic conditions defined by Elixhauser Comorbidities [43] (shown in Table 2). Sensitivity analyses involved measuring a claims-based frailty index [44].

### Statistical analyses

The sociodemographic and clinical characteristics of patients were described for those who were and were not prescribed a potentially inappropriate medication during the 90-day follow-up. To facilitate interpretation, some continuous variables were categorized based on quartiles for ambulatory visits and percentiles for inpatient stays, emergency department visits, and non-acute stays. We provided absolute standardized differences, which are the differences in the mean of a covariate between two groups, to show any differences between groups [45].

Cox regression analysis was conducted to estimate the effect of baseline demographic and clinical characteristics on the probability of being prescribed a new PIM during the 90-day follow-up period. We chose Cox regression analysis in particular as the primary analysis to better model the likelihood of prescribing as a function of time. In specific, we computed hazard ratios (HR) and 95% confidence intervals (CI). First, we tested each variable separately, without adjusting for other covariates, using Cox proportional hazards regression analysis to estimate its association with the new prescribing of potentially inappropriate medications. Next, we used multivariable Cox proportional hazards regression analysis to examine the association between each variable and the prescribing of PIMs, while adjusting for other covariates. In the first multivariable model, we included demographic and healthcare utilization variables. In the second one, we added information on the types of chronic conditions. The first encounter in which a PIM was prescribed was analyzed in the time-to-event analyses. Finally, we analyzed the types of PIMs prescribed in the follow-up period.

We also performed some subgroup (e.g., restricted to patients with ≥1 prescribing order in the baseline period, with ≥2 ambulatory visits in the baseline period) and sensitivity analyses (e.g., exclude Beers criteria with a low level of evidence, adding a claims-based frailty index to the model, extending the baseline period to 365 days, and keeping all continuous variables in their original form). In addition, we performed a multivariable logistic regression with the same outcome and same variables in the model. We performed all analyses using STATA 15.1 (StataCorp, College Station, TX, USA). Statistical significance was determined by using two-sided tests with an α of 0.05.

This study was approved by the Brigham and Women’s Hospital Institutional Review Board. We followed the reporting requirements of the ‘Strengthening the Reporting of Observational Studies in Epidemiology’ (STROBE) guidelines [46].

## Results

In total, we identified 17,912 older adults with multimorbidity and polypharmacy who were naïve to a potentially inappropriate medication in the prior 180 days and met all other inclusion and exclusion criteria (Fig. 1). Of these, 447 (2.5%) were prescribed a new potentially inappropriate medication during the 90-day follow-up period. Central nervous system drugs, cardiovascular drugs, anticholinergics, and endocrine drugs were the most commonly prescribed PIMs (Table 1). Benzodiazepines were prescribed in 29% of patients who were newly prescribed a central nervous system PIM.

**Table 1:** Potentially inappropriate medications (PIMs) prescribed during the 90-day follow-up period (*N* = 17,912)

*Types of potentially inappropriate medications*	*Potentially inappropriate medications*
	*Number of patients* *(% of total number of patients with PIM prescription)*
All	447 (2.5% of patients in cohort)
Anticholinergics	64 (14.3)
First-generation antihistamines	52 (11.6)
Antiparkinsonian agents	**
Antispasmodics	**
*Anti-infective* (Nitrofurantoin)	**
Cardiovascular	90 (20.1)
Peripheral alpha-1 blockers	14 (3.1)
Central alpha agonists	**
Disopyramide	**
Digoxin	57 (12.8)
Nifedipine	**
Amiodarone	21 (4.7)
Central nervous system	185 (41.4)
Antidepressants	**
Antipsychotics	35 (7.8)
Barbiturates	**
Benzodiazepines	130 (29.1)
Nonbenzodiazepine	29 (6.5)
Endocrine	77 (17.2)
Androgens	**
Growth hormone	16 (3.6)
Insulin	39 (8.7)
Megestrol	**
Sulfonylureas	16 (3.6)
Gastrointestinal	20 (4.5)
Metoclopramide	12 (2.7)
Mineral oil	**
Proton-pump inhibitors	**
Pain medications	54 (12.1)
Non-cycloocygenase-selective NSAIDs	17 (3.8)
Indomethacin, ketorolac	20 (4.5)
Skeletal muscle relaxants	18 (4.0)

** cells < 11 suppressed for data protection reasons according to Medicare requirements

*Not presented due to not having been prescribed during the 90-day follow-up period: Antithrombotics* (Dipyridamole), Dronedrone, Meprobamate, Ergoloid mesylates, Desiccated thyroid Estrogens, Meperidine, Genitourinary (Desmopressin)

Table 2 shows the baseline characteristics of all patients overall and by whether they were newly-prescribed a PIM. In the entire cohort, the average age was 78 years (SD = 7.5), and 58.6% of patients were female. Patients newly-prescribed a PIM differed from those who were not prescribed a PIM; for example, new PIM prescribing was higher among men and those with greater prior healthcare services use. The types of chronic conditions were relatively comparable between the two groups at baseline, except for congestive heart failure.

**Table 2:** Baseline characteristics, by whether the participants had a new prescription of a potentially inappropriate medication (PIM) during the 90-day follow-up period

	*All patients*	*Patients with new PIM prescription during follow-up*	*Patients without new PIM prescription during follow-up*	*Absolute standardized differences*^*d*^
	*(n = 17,912)*	*(n = 447)*	*(N = 17,465)*	
Demographic characteristics				
Mean age in years (SD)	78.0 (7.5)	77.0 (7.5)	78.0 (7.5)	0.13
Female sex (%)	10,497 (58.6)	225 (50.3)	10,272 (58.8)	0.17
Hispanic ethnicity (%)	161 (0.9)	**	**	0.04
Race (%)				
White	16,593 (92.6)	398 (89.0)	16,195 (92.7)	0.13
Black	653 (3.7)	24 (5.4)	629 (3.6)	
Asian	165 (0.9)	**	**	
Other	417 (2.3)	17 (3.8)	400 (2.3)	
No information	84 (0.5)	**	**	
Medication intake				
Mean (SD) drug items dispensed per patient with min. 90 days’ supply	6.1 (1.4)	6.2 (1.4)	6.1 (1.4)	0.07
Mean (SD) drug items dispensed	8.0 (2.4)	8.2 (2.5)	8.0 (2.4)	0.08
Healthcare utilization				
Patients with at least 1 inpatient stay (%)	3001 (16.7)	97 (21.7)	2904 (16.6)	0.13
Patients with at least 1 emergency department visit (%)	4711 (26.3)	143 (32.0)	4568 (26.2)	0.13
Patients with at least 1 non-acute institutional stay (%)	2360 (13.2)	45 (10.1)	2315 (13.3)	0.10
Patients with number of ambulatory visits above the median number of ambulatory visits (median = 17) (%)^b^	9186 (51.3)	293 (65.6)	8893 (50.9)	0.30
Number of chronic conditions				
Mean (SD) number of chronic conditions	5.1 (2.3)	5.4 (2.5)	5.1 (2.3)	0.12
Chronic conditions types^a^ (%)				
Congestive heart failure	2931 (16.4)	103 (23.0)	2828 (16.2)	0.17
Cardiac arrhythmias	1061 (5.9)	37 (8.3)	1024 (5.9)	0.10
Valvular disease	2109 (11.8)	56 (12.5)	2053 (11.8)	0.02
Pulmonary circulation disorders	399 (2.2)	83 (18.6)	2748 (15.7)	0.08
Peripheral vascular disorders	2525 (14.1)	70 (16.7)	2455 (14.1)	0.05
Hypertension	11,309 (63.1)	285 (63.8)	11,024 (63.1)	0.01
Chronic pulmonary disorders	2831 (15.8)	83 (18.6)	2748 (15.7)	0.08
Diabetes	5718 (31.9)	161 (36.0)	5557 (31.8)	0.09
Hypothyroidism	2531 (14.1)	59 (13.2)	2472 (14.2)	0.03
Renal failure	1669 (9.3)	46 (10.3)	1623 (9.3)	0.03
Liver disease	274 (1.5)	**	**	0.08
Cancer	2447 (13.7)	67 (15.0)	2380 (13.6)	0.04
Rheumatoid arthritis / collagen vascular diseases	940 (5.3)	20 (4.5)	920 (5.3)	0.04
Coagulopathy	619 (3.5)	19 (4.3)	600 (3.4)	0.04
Fluid and electrolyte disorders	783 (4.4)	25 (5.6)	758 (4.3)	0.06
Psychoses	309 (1.7)	**	**	0.05
Depression	1580 (8.8)	38 (8.5)	1542 (8.8)	0.01
Other characteristics				
Mean frailty index (SD)^c^	0.16 (0.1)	0.16 (0.1)	0.16 (0.1)	0.04

Missing data < 2% for all variables listed. ^a^comorbidities defined with coding algorithms for defining Elixhauser comorbidities in ICD-9 administrative data (Quan et al. 2005), ≥2 ICD-9 codes per category, hypertension categories merged, diabetes categories merged, different cancer categories merged, drug abuse, alcohol abuse, obesity, weight loss, HIV/AIDS, paralysis, other neurological disorders, blood loss anemia and deficiency anemia not presented; ^b^due to our cohort definition all patients had min. 1 ambulatory visit during the baseline period; ^c^Kim DH, Schneeweiss S, Lipsitz LA, Glynn R, Rockwood K, Avorn J. Measuring Frailty in Medicare Data: Development and Validation of a Claims-Based Frailty Index. J Gerontol A Biol Sci Med Sci. 2018; 73: 980–987. doi: 10.1093/gerona/glx229. PMID: 29244057; PMCID: PMC6001883; ^d^ A significant difference between the proportions of patients is usually characterized by an absolute standardized difference ≥ ±0.1; **cells < 11 suppressed for data protection reasons according to Medicare requirements

Unadjusted and multivariable Cox regression of the association between measured patient factors and the risk of being prescribed a PIM in older multimorbid patients with polypharmacy are shown in Table 3. Of note, there were no violations of the proportional hazards’ assumption. In unadjusted analyses, increased age (i.e. ≥85 years), male sex, and some racial groups (e.g. Black) were associated with being newly-prescribed a potentially inappropriate medication (Table 3). Most variables measuring the health services use of patients, such as the number of inpatient stays, number of emergency department visits, number of ambulatory visits, and the number of prescribing orders were associated with an increased risk of PIM prescribing. The number of chronic conditions and some types of chronic conditions (i.e. congestive heart failure, cardiac arrhythmias) were also associated with new PIM prescriptions.

**Table 3:** Unadjusted and multivariable associations between demographic and clinical factors and the prescribing of potentially inappropriate medications during the 90-day follow-up period

*Demographics and clinical characteristics*	*Unadjusted associations*		*Model 1: Demographic and healthcare utilization variables*		*Model 2: Model 1 + chronic conditions*	
	*Unadjusted hazard ratio*	*95% CI*	*Adjusted hazard ratio*	*95% CI*	*Adjusted hazard ratio*	*95% CI*
	(*N* = 17,912)		(*n* = 17,911)		(*n* = 17,911)	
Age (reference: 65–74)						
75–84	0.85	0.69–1.04	0.86	0.70–1.06	0.87	0.71–1.07
85 and above	0.67	0.52–0.88**	0.76	0.58–0.99**	0.75	0.56–0.99**
Male sex (reference: female sex)	1.41	1.17-1.79***	1.30	1.08–1.57**	1.29	1.06–1.57**
Hispanic ethnicity (reference: non-hispanic)	1.51	0.67–3.38	0.96	0.35–2.59	0.95	0.35–2.67
Race (reference: White)						
Asian	1.26	0.52–3.03	1.37	0.57–3.31	1.31	0.54–3.17
Black	1.54	1.02–2.33**	1.54	1.02–2.33**	1.50	0.98–2.27
Other	1.71	1.05–1.78**	1.64	0.90–2.99	1.59	0.87–2.90
Number of inpatient stays (reference: 0^b^)						
At least 1	1.39	1.11–1.75**	0.98	0.72–1.33	0.94	0.69–1.28
Number of emergency department visits (reference: 0^c^)						
At least 1	1.33	1.09–1.62**	1.12	0.86–1.33	1.09	0.84–1.41
Number of ambulatory visits (reference: ≤9^d^)						
10–17	0.94	0.70–1.28	0.96	0.70–1.31	0.96	0.70–1.31
18–29	1.39	1.05–1.84**	1.42	1.05–1.91**	1.42	1.06–1.92**
≥ 30	2.12	1.64–2.76***	2.12	1.55–2.93**	2.12	1.53–2.95**
Number of non-acute institutional stays (reference: 0^e^)						
At least 1	0.74	0.55–1.01	0.81	0.58–1.13	0.76	0.54–1.08
Level of polypharmacy (reference: 5–9 medications)						
10 and above	1.27	0.77–2.09	1.16	0.70–1.92	1.08	0.65–1.79
Number of chronic conditions (1-unit increase)	1.06	1.02–1.10**	0.96	0.91–1.01	0.94	0.88–1.01
Number of prescribing orders (1-unit increase)	1.02	1.01–1.02***	1.02	1.01–1.02***	1.02	1.01–1.02***
Types of chronic conditions^a^						
Congestive heart failure	1.56	1.25–1.94***	–	–	1.38	1.07–1.78**
Cardiac arrhythmias	1.44	1.03–2.02**	–	–	1.02	0.71–1.46
Valvular disease	1.08	0.82–1.43	–	–	0.85	0.63–1.15
Pulmonary circulation disorders	1.64	0.99–2.71	–	–	1.41	0.84–2.38
Peripheral vascular disorders	1.14	0.88–1.47	–	–	1.09	0.84–1.42
Hypertension	1.03	0.85–1.25	–	–	0.98	0.78–1.22
Chronic pulmonary disorders	1.23	0.97–1.56	–	–	1.10	0.85–1.43
Diabetes	1.20	0.99–1.46	–	–	1.15	0.93–1.43
Hypothyroidism	0.92	0.70–1.21	–	–	1.08	0.81–1.45
Renal failure	1.13	0.83–1.53	–	–	0.90	0.65–1.25
Cancer	1.12	0.86–1.45	–	–	0.88	0.66–1.17
Rheumatoid arthritis/collagen vascular diseases	0.85	0.54–1.33	–	–	0.81	0.51–1.28
Coagulopathy	1.25	0.79–1.98	–	–	0.95	0.58–1.54
Fluid and electrolyte disorders	1.30	0.87–1.95	–	–	1.11	0.73–1.68
Depression	0.97	0.69–1.35	–	–	1.04	0.73–1.48
Liver disease	1.81	1.02–3.20	–	–	1.54	0.86–2.75

Overall follow-up time in days: 1,575,994; average follow-up time in days: 88. ^a^ comorbidities defined with coding algorithms for defining Elixhauser comorbidities in ICD-9 administrative data (Quan et al. 2005), ≥2 ICD-9 codes per category, hypertension categories merged, diabetes categories merged, different cancer categories merged, drug abuse, alcohol abuse, obesity, weight loss, HIV/AIDS, paralysis, other neurological disorders, blood loss anemia and deficiency anemia not included, ^b^ 90th percentile = 1, ^c^ 75th percentile = 1. ^d^ categories based on quartiles, ^e^85th percentile = 1

** *p* < 0.05; *** *p* < 0.001

In the multivariable analysis including demographic and healthcare utilization variables, male sex, Black race, ≥85 years of age, number of ambulatory visits (≥18 visits during the baseline period), and number of prescribing orders were associated with new PIM prescribing. In model 2, including chronic conditions, we observed similar results. In this model, male sex (adjusted hazard ratio (HR) = 1.29; 95%CI: 1.06–1.57), age (≥85 years: HR = 0.75, 95%CI: 0.56–0.99, 75–84 years: HR = 0.87, 95%CI: 0.71–1.07; reference: 65–74 years), number of ambulatory visits (18–29 visits: HR = 1.42, 95%CI: 1.06–1.92; ≥30 visits: HR = 2.12, 95%CI: 1.53–2.95, reference: ≤9 visits), the number of prescribing orders (HR = 1.02 per 1-unit increase, 95%CI: 1.01–1.02), and a heart failure diagnosis (HR = 1.38, 95%CI: 1.07–1.78) were associated with being newly-prescribed a PIM, but Black race was no longer significantly associated with new PIM prescribing.

Extending the baseline period to 365  days (eTable 2) and analyzing all continuous variables as continuous variables (eTable 3) did not change the results, and there were similar results using multivariable logistic regression (eTable 4). Similar results were also observed when restricting the analyses to Beers criteria with moderate or high level of evidence (eTable 5). When analyzing patients with ≥3 chronic conditions (eTable 6) or those with ≥2 ambulatory visits during the baseline period (eTable 7), we found however that age (≥85 years) and Black race where no longer significantly associated with new PIM prescribing. Age was also no longer significantly associated when adding a claims-based frailty index to the model (eTable 8).

## Discussion

This is the first study exploring factors associated with new prescribing of potentially inappropriate medications in older multimorbid adults with polypharmacy in a US sample without prior PIM use. Of the 2.5% of patients who were newly prescribed a PIM within the follow-up period of 90 days, male sex, more ambulatory visits, more prescriptions, and prior diagnosis of heart failure were associated with new receipt of a PIM prescription and being ≥85 years of age was associated with a lower risk of new PIM prescribing. Central nervous system drugs, cardiovascular drugs, anticholinergics, and endocrine drugs were the most commonly prescribed PIMs. The finding that benzodiazepines was the most commonly prescribed PIM is in line with previous research [21, 47].

Of the patient demographic factors found to be associated with new PIM prescribing, male sex, and Black race were found to be associated with an increased risk of a new prescription for a PIM, while advanced age was found to be associated with an decrease risk of a new PIM prescription. Prior literature is inconclusive on whether sex is associated with the prescribing of PIMs. While some studies found an association with female sex and PIM prescribing [22, 48], others did not find any significant association [21, 49, 50]. Most previous studies were cross-sectional, which does not provide evidence about the incidence of new PIM prescribing and factors associated with it.

Our results were mixed on whether there is an association between the new prescribing of PIMs and age. In our main model, we observed that those ≥85 years had a lower risk of new PIM prescribing, but this was no longer significant in some sensitivity analyses. Similarly, while some previous studies did not observe any association, others also found a protective factor of age [48, 51]. Furthermore, when examining Black race we found mixed results, as Black race was no longer significantly associated with PIM prescribing after adjusting for the types of chronic conditions. Prior research in more general populations of older adults has, in fact, found a positive association between white race and greater PIM prescribing [52, 53].

We found that an above median number of ambulatory care visits and the number of prescribing orders were positively associated with PIM prescribing, which could be indicators of clinical complexity. Current literature on PIM prescribing and health services use is mixed: one study found an association between inpatient stays, emergency department visits and outpatient visits [54], while another one did not find an association between PIM prescribing and outpatient visits [50]. There is some evidence that PIM prescribing may be positively associated with the number of prescribers [21]. Contrasted with patient demographic and health services use factors, the presence of specific chronic comorbidities was generally not found to be associated with new PIM prescribing. This is in line with previous research [22].

Overall, we observed that patients who were newly prescribed a PIM during the follow-up period were slightly sicker, had a higher health services use, and thus were more complex. This increased clinical complexity could lead to less oversight by individual providers on patients’ medication regimens, which in turn could make the prevention and reduction of PIM prescribing more difficult. We hypothesize that rather being a question of the individual factors associated with new PIM prescribing, the complexity of individual patients their treatment schedules and medication regimens could be strongly associated with greater PIM prescribing.

These findings have several implications for clinical care. Healthcare professionals, such as pharmacists and physicians, should be aware of key demographic factors that appear to be associated with PIM prescribing when taking prescribing decisions and potentially incorporate these into decision support for prescribers. Relatedly, improvement of care coordination across providers and fragmentation of healthcare prescribing decisions may also be critical ingredients for reducing PIM prescribing in this population, given that more ambulatory visits and unique prescribing orders are also associated with receiving PIMs.

Further, these findings have implications for the design of interventions aimed at reducing the prescribing of PIMs and deprescribing interventions. Prior medication optimization interventions in older adults more broadly have had little or no effect on clinical outcomes, such as mortality or cognitive impairment [55]. Current evidence on interventions in multimorbid older adults using multiple medications remains scarce [56]. Interventions designed to optimize prescribing also may need to be multifaceted, as they should aim at changing behaviors of different stakeholders (e.g. patients, physicians, pharmacists, etc.) and should involve different components (i.e. medication review, education/training, and use different tools/instruments) [57, 58]. Such interventions must not only solve medication-related problems (e.g. PIM prescribing), but they must target the underlying mechanisms that lead to these problems (e.g. complexity). Consequently, while not all of the above-mentioned factors are modifiable, the knowledge of their association with PIM prescribing must be built into medication optimization interventions and may be even more important for this more complex population.

### Limitations

While to the best of our knowledge this study was the first to examine patient factors for new prescribing of PIMs in older multimorbid adults with polypharmacy, there are several limitations. First, the data are from several years ago (owing, in part, to an administrative lag in Medicare data and linking with EHR data); however, prescribing rates of PIMs have not changed since 2007-2014 [59]. Consequently,  we expect the exploration of risk factors to remain highly relevant. Second, due to the criterion-based rather than judgment-based nature of the Beers list, medications indicated in certain circumstances (e.g. use as last resort, etc.) may have been flagged as potentially inappropriate. Despite this, the Beers criteria are the most commonly used tool for defining PIM use in the US and restricting the analyses to medications with medium and high level of evidence did not change the results. Despite our data covering the period from 2007 to 2014, we used the 2019 Beers list to inform current medical decision making. This comes with a modest limitation that medications that were included in the Beers list were excluded in the meantime and some new ones were added. Overall, however, these changes only concerned a small number of medications listed in Table 2 of the Beers criteria (e.g., in the 2015 version of the Beers criteria, four medications were removed and three were added; in the 2019 version of the Beers criteria, two medications were removed and three medications were added) [38, 39]. Furthermore, we were not able to adjust our models for the number and types of prescribers, but we specifically focused on patient factors for this reason. Third, we had limited information on dose and route of administration of medications, which may have affected the definition of PIMs; however, this might have led to an underestimation of the prescribing of gastrointestinal PIMs. We also may have underestimated the new prescribing of PIMs because we may not have captured over-the-counter prescribing of medications (e.g. anti-histamines). Fourth, despite the demographic makeup of the Boston metropolitan area being similar to other urban US regions [60], access to healthcare and physicians may be higher in this area compared to other parts of the country. The present study is an observational study, so residual confounding cannot be excluded because of unmeasured or inadequately measured confounders. Finally, there may have been selection bias, since the patients who achieve a high age without being prescribed or using a PIM may differ from those with PIM prescribing or use.

## Conclusion

Several demographic and clinical characteristics are associated with the new prescribing of potentially inappropriate medications in older patients who were naïve to PIMs (e.g. age of ≥85 years, male sex, and number of ambulatory visits). This also indicates that patients with more complex health problems may be at a higher risk of new PIM prescribing. Central nervous system drugs, cardiovascular drugs, anticholinergics and endocrine drugs were the most commonly prescribed PIMs during the 90-day follow-up period. Due to the potential negative outcomes associated with the use of PIMs, these study findings should inform the creation of interventions to improve coordination of care and reduce the prescribing of potentially inappropriate medications in older multimorbid adults with polypharmacy.

## Supplementary Information

**Additional file 1.**

## Data Availability

The data that support the findings of this study are available from the Research Data Assistance Center (ResDAC) from the Centers for Medicare and Medicaid Services and from Mass General Brigham (formerly Partners Healthcare). Restrictions apply to the availability of these data, which were used under license for the current study, and so are not publicly available. Data may however be available from the authors upon reasonable request and with permission of ResDAC.

## Abbreviations

AGS: American Geriatrics Society
AHRQ: Agency for Healthcare Research and Quality
CCI: Chronic Condition Indicator
CMS: Centers for Medicare and Medicaid Services
EHR: Electronic health records
FDA: Food and Drug Administration
PIMs: Potentially inappropriate medications
RPDR: Partners Research Patient Data Registry
STROBE: Strengthening the Reporting of Observational Studies in Epidemiology
