# Supplementary material for: Patient factors associated with new prescribing of potentially inappropriate medications in multimorbid US older adults using multiple medications
Source: BMC Geriatr. 2021 Mar 6;21:163. doi: 10.1186/s12877-021-02089-x (PMC7937195; doi:10.1186/s12877-021-02089-x)
Supplement: Supplementary file 1 — Additional file 1. [file 12877_2021_2089_MOESM1_ESM.docx]

**Supplementary Material**

**Patient factors associated with new prescribing of potentially inappropriate medications in multimorbid US older adults using multiple medications**

Katharina Tabea Jungo, MSc^1,2,3,4^, ORCID: 0000-0002-1782-1345

Sven Streit, MD, MSc, PhD^1^, ORCID: 0000-0002-3813-4616

Julie C Lauffenburger, PharmD, PhD^3,5^, ORCID: 0000-0002-4940-4140

^1^ Institute of Primary Health Care (BIHAM), University of Bern, Bern, Switzerland.

^2^ Department of Epidemiology, Harvard T.H. Chan School of Public Health, Boston, Massachusetts.

^3^ Division of Pharmacoepidemiology and Pharmacoeconomics, Department of Medicine, Brigham and Women's Hospital and Harvard Medical School, Boston, Massachusetts.

^4^ Graduate School for Health Sciences, University of Bern, Switzerland

^5^ Center for Healthcare Delivery Sciences, Department of Medicine, Brigham and Women's Hospital, Boston, Massachusetts.

**Table of Content**

[eTable 1. Beers criteria included in the analyses 2](#_Toc58255218)

[eTable 2. Cohort with 365 days baseline period (N = 22,072): Multivariable associations between demographic and clinical factors and the prescribing of potentially inappropriate medications (PIMs) 5](#_Toc58255219)

[eTable 3. Multivariable associations between demographic and clinical factors (continuous variables) and the prescribing of potentially inappropriate medications (PIMs) 7](#_Toc58255220)

[eTable 4. Unadjusted and multivariable associations between demographic and clinical factors and the prescribing of potentially inappropriate medications (logistic regression) 9](#_Toc58255221)

[eTable 5. Multivariable associations between demographic and clinical factors and the prescribing of potentially inappropriate medications (PIMs) with moderate or high levels of evidence* 11](#_Toc58255222)

[eTable 6. Multivariable associations between demographic and clinical factors and the prescribing of potentially inappropriate medications in patients with ≥3 chronic conditions 13](#_Toc58255223)

[eTable 7. Multivariable associations between demographic and clinical factors and the prescribing of potentially inappropriate medications in patients with min. 2 ambulatory visits recorded in the electronic medical records during the baseline period 15](#_Toc58255224)

[eTable 8. Multivariable associations between demographic and clinical factors and the prescribing of potentially inappropriate medications in patients: Sensitivity analysis with claims-based frailty index 17](#_Toc58255225)

# eTable 1. Beers criteria included in the analyses

| **Medications** | **Comments** |
| --- | --- |
| *Anticholinergics* | |
| First-generation antihistamines | medications flagged as potentially inappropriate as listed in table 2 of the 2019 Beers criteria |
| Antiparkinsoinian agents | medications flagged as potentially inappropriate as listed in table 2 of the 2019 Beers criteria |
| Antispasmodics | medications flagged as potentially inappropriate as listed in table 2 of the 2019 Beers criteria |
| *Antithrombotics* | |
| Dipyridamole | medications flagged as potentially inappropriate as listed in table 2 of the 2019 Beers criteria |
| *Anti-infective* | |
| Nitrofurantoin | flagged as potentially inappropriate when Nitrofurantoin used for ≥90days or with min. 2 refills or in individuals with creatinine clearance <30mL/min |
| *Cardiovascular* | |
| Peripheral alpha-1 blockers for treatment of hypertension | flagged as potentially inappropriate when preceded by hypertension diagnosis |
| Central alpha agonists, other CNS alpha-agonists | Clonidine flagged as potentially inappropriate when preceded by hypertension diagnosis, other CNS alpha-agonists as listed in table 2 flagged as potentially inappropriate |
| Disopyramide | medication flagged as potentially inappropriate as listed in table 2 of the 2019 Beers criteria |
| Dronedarone | Flagged as potentially |
| Digoxin | Flagged as potentially inappropriate medication when used after previous hypertension diagnosis, when used after heart failure diagnosis |
| Nifedipine | Medication flagged as potentially inappropriate as listed in table 2 of the 2019 Beers criteria |
| Amiodarone | Flagged as potentially inappropriate medication when used after atrial fibrillation therapy, unless patient has heart failure or substantial left ventricular hypertrophy |
| *continued* | |

| **Medications** | **Comments** |
| --- | --- |
| *Central nervous system* | |
| Antidepressants, alone or in combination | Medication flagged as potentially inappropriate as listed in table 2 of the 2019 Beers criteria |
| Antipsychotics, first and second generation | Flag as potentially inappropriate, unless patient has schizophrenia, bipolar disorder, or for short-term use as antiemetic during chemotherapy |
| Barbiturates | Medications flagged as potentially inappropriate as listed in table 2 of the 2019 Beers criteria |
| Benzodiazepines | Medications flagged as potentially inappropriate as listed in table 2 of the 2019 Beers criteria |
| Meprobamate | Medication flagged as potentially inappropriate as listed in table 2 of the 2019 Beers criteria |
| Z-drugs | Medications flagged as potentially inappropriate as listed in table 2 of the 2019 Beers criteria |
| Ergoloid mesylates | Medication flagged as potentially inappropriate as listed in table 2 of the 2019 Beers criteria |
| *Endocrine* | |
| Androgens | Flagged as potentially inappropriate unless there is a hypogonadism diagnosis |
| Desiccated thyroid | Medication flagged as potentially inappropriate as listed in table 2 of the 2019 Beers criteria |
| Estrogens | Flagged as potentially inappropriate when oral or topical patch, vaginal cream and vaginal tablets not flagged as potentially inappropriate |
| Growth hormone | Flagged as potentially inappropriate |
| Insulin | Flagged as potentially inappropriate when only short- or rapid-acting insulins without combination with long-acting insulins |
| Megesterol | Medication flagged as potentially inappropriate as listed in table 2 of the 2019 Beers criteria |
| Sulfonylureas | Medications flagged as potentially inappropriate as listed in table 2 of the 2019 Beers criteria |
| *Gastrointestinal* | |
| Metoclopramide | Flagged as potentially inappropriate unless there is a diagnosis of gastroparesis and not used for longer than 84 days or min. 2 refills |
| *continued* | |

| **Medications** | **Comments** |
| --- | --- |
| *Gastrointestinal (continued)* | |
| Mineral oil | Flagged as potentially inappropriate when given orally |
| Proton-pumps inhibitors | Flagged as potentially inappropriate when used for ≥ 56 days or min. 2 refills without any of the mentioned diagnoses (e.g., esophagitis, hypersecretory condition), or when used for ≥56 days or min. 2 refills not preceded by chronic corticosteroid or NSAID use (≥90 days or min. 2 refills) |
| *Pain medications* | |
| Meperidine | Medication flagged as potentially inappropriate as listed in table 2 of the 2019 Beers criteria |
| non-cyclooxygenase-selective NSAIDs | Medications flagged as potentially inappropriate as listed in table 2 of the 2019 Beers criteria. Flagged as potentially inappropriate when used for ≥ 90 days or min. 2 refills and when there is no overlapping PPI or misoprostol use |
| Indomethacin, Ketorolac | Medications flagged as potentially inappropriate as listed in table 2 of the 2019 Beers criteria |
| Skeletal muscle relaxants | Medications flagged as potentially inappropriate as listed in table 2 of the 2019 Beers criteria |
| *Genitourinary* | |
| Desmopressin | Medications flagged as potentially inappropriate as listed in table 2 of the 2019 Beers criteria |
| Reference: (2019), American Geriatrics Society 2019 Updated AGS Beers Criteria® for Potentially Inappropriate Medication Use in Older Adults. J Am Geriatr Soc, 67: 674-694. doi:10.1111/jgs.15767 – table 2. | |

# eTable 2. Cohort with 365 days baseline period (N = 22,072): Multivariable associations between demographic and clinical factors and the prescribing of potentially inappropriate medications (PIMs)

| Demographics and clinical characteristics | | | | Model 1: Demographic and healthcare utilization variables | | Model 2: Model 1 + chronic conditions | |
| --- | --- | --- | --- | --- | --- | --- | --- |
|  | | | | Adjusted hazard ratio | 95% CI | Adjusted hazard ratio | 95% CI |
|  | | | | (n = 22,071) | | (n = 22,071) | |
| Age (reference: 65-74) | | | |  | | | |
|  | 75-84 | | | 0.85 | 0.70-1.05 | 0.88 | 0.72-1.07 |
|  | 85 and above | | | 0.80 | 0.62-1.03 | 0.83 | 0.64-1.07 |
| Male sex (reference: female sex) | | | | 1.29 | 1.08-1.55** | 1.28 | 1.06-1.53** |
| Hispanic ethnicity (reference: non-hispanic) | | | | 1.23 | 0.51-3.00 | 1.23 | 0.51-2.99 |
| Race (reference: White) | | | |  | | | |
|  | | | Asian | 2.02 | 1.00-4.06** | 1.88 | 0.93-3.80 |
|  | | | Black | 1.52 | 1.02-2.27** | 1.42 | 0.95-2.11 |
|  | | | Other | 1.52 | 0.87-2.65 | 1.43 | 0.82-2.51 |
| Number of inpatient stays (reference: 0) | | | |  | | | |
|  | | 1 or above | | 1.08 | 0.83-1.42 | 1.07 | 0.81-1.42 |
| Number of emergency department visits (reference: 0) | | | |  | | | |
|  | | | 1 or above | 1.06 | 0.83-1.34 | 1.04 | 0.82-1.32 |
| Number of ambulatory visits (reference: ≤11) | | | | | | | |
|  | 12-20 | | | 1.00 | 0.75-1.33 | 1.01 | 0.75-1.34 |
|  | 21-36 | | | 1.47 | 1.12-1.92”” | 1.50 | 1.14-1.97** |
|  | ≥37 | | | 1.81 | 1.33-2.47*** | 1.86 | 1.36-2.55*** |
| Number of non-acute institutional stays (reference: 0) | | | | | | | |
|  | 1 or above | | | 0.89 | 0.65-1.21 | 0.87 | 0.64-1.20 |
| Level of polypharmacy (reference: 5-9 medications) | | | | | | | |
|  | 10 and above | | | 1.53 | 1.01-2.31 | 1.43 | 0.94-2.17 |
| Number of chronic conditions (1-unit increase) | | | | 0.95 | 0.91-0.99** | 0.94 | 0.89-0.99** |
| Number of prescribing orders (1-unit increase) | | | | 1.01 | 1.01-1.02*** | 1.01 | 1.01-1.02*** |
| continued | | | | | | | |

| Demographics and clinical characteristics | | Model 1: Demographic and healthcare utilization variables | | Model 2: Model 1 + chronic conditions | |
| --- | --- | --- | --- | --- | --- |
|  | | Adjusted hazard ratio | 95% CI | Adjusted hazard ratio | 95% CI |
| Types of comorbidities^1^ | |  | | | |
|  | Congestive heart failure | - | - | 1.05 | 0.81-1.35 |
|  | Cardiac arrhythmias | - | - | 1.06 | 0.76-1.46 |
|  | Valvular disease | - | - | 0.97 | 0.74-1.27 |
|  | Pulmonary circulation disorders | - | - | 1.63 | 1.04-2.56 |
|  | Peripheral vascular disorders | - | - | 0.99 | 0.78-1.27 |
|  | Hypertension | - | - | 0.97 | 9,,78-1.21 |
|  | Chronic pulmonary disorders | - | - | 1.08 | 0.85-1.38 |
|  | Diabetes | - | - | 1.28 | 1.05-1.55** |
|  | Hypothyroidism | - | - | 0.89 | 0.67-1.16 |
|  | Renal failure | - | - | 1.01 | 0.75-1.37 |
|  | Cancer | - | - | 0.85 | 0.64-1.10 |
|  | Rheumatoid arthritis/collagen vascular diseases | - | - | 0.98 | 0.66-1.45 |
|  | Coagulopathy | - | - | 0.95 | 0.60-1.52 |
|  | Fluid and electrolyte disorders | - | - | 0.97 | 0.75-1.45 |
|  | Depression | - | - | 1.08 | 0.79-1.49 |
|  | Liver disease | - | - | 1.25 | 0.70-2.23 |
| ^1^ comorbidities defined with coding algorithms for defining Elixhauser comorbidities in ICD-9 administrative data (Quan et al. 2005), ≥2 ICD-9 codes per category, hypertension categories merged, diabetes categories merged, different cancer categories merged, drug abuse, alcohol abuse, obesity, weight loss, HIV/AIDS, paralysis, other neurological disorders, blood loss anemia and deficiency anemia not included;  *PIMs with low level of evidence excluded.  ** p<0.05; *** p<0.001 | | | | | |

# eTable 3. Multivariable associations between demographic and clinical factors (continuous variables) and the prescribing of potentially inappropriate medications (PIMs)

| Demographics and clinical characteristics | | Model 1: Demographic and healthcare utilization variables | | Model 2: Model 1 + chronic conditions | |
| --- | --- | --- | --- | --- | --- |
|  | | Adjusted hazard ratio | 95% CI | Adjusted hazard ratio | 95% CI |
|  | | (n = 17,911) | | (n = 17,911) | |
| Age (per 10-year increase) | | 0.89 | 0.78-1.02 | 0.88 | 0.77-1.01 |
| Male sex (reference: female sex) | | 1.31 | 1.08-1.58** | 1.29 | 1.06-1.57** |
| Hispanic ethnicity (reference: non-hispanic) | | 1.00 | 0.37-2.72 | 0.98 | 0.362.67 |
| Race (reference: White) | |  | | | |
|  | Asian | 1.34 | 0.56-3.25 | 1.29 | 0.53-3.13 |
|  | Black | 1.58 | 1.04-2.39** | 1.53 | 1.01-2.33** |
|  | Other | 1.54 | 0.84-2.82 | 1.52 | 0.83-2.79 |
| Number of inpatient stays (1-unit increase) | | 0.87 | 0.76-1.00 | 0.86 | 0.74-0.99** |
| Number of emergency department visits (1-unit increase) | | 0.99 | 0.98-1.02 | 0.99 | 0.97-1.02 |
| Number of ambulatory visits (10-unit increase) | | 1.01 | 1.00-1.02*** | 1.01 | 1.00-1.02 |
| Number of non-acute institutional stays (1-unit increase) | | 0.99 | 0.98-1.01 | 0.99 | 0.97-1.01 |
| Number of chronic medications (1-unit increase) | | 1.04 | 0.97-1.11 | 1.01 | 0.94-1.09 |
| Number of chronic conditions (1-unit increase) | | 0.99 | 0.94-1.04 | 0.97 | 0.90-1.04 |
| Number of prescribing orders (1-unit increase) | | 1.02 | 1.01-1.02*** | 1.02 | 1.01-1.02*** |
|  | |  |  |  | continued |

| Demographics and clinical characteristics | | Model 1: Demographic and healthcare utilization variables | | Model 2: Model 1 + chronic conditions | |
| --- | --- | --- | --- | --- | --- |
|  | | Adjusted hazard ratio | 95% CI | Adjusted hazard ratio | 95% CI |
| Types of comorbidities^1^ | |  | | | |
|  | Congestive heart failure | - | - | 1.42 | 1.10-1.83** |
|  | Cardiac arrhythmias | - | - | 1.09 | 0.76-1.56 |
|  | Valvular disease | - | - | 0.87 | 0.64-1.17 |
|  | Pulmonary circulation disorders | - | - | 1.45 | 0.86-2.44 |
|  | Peripheral vascular disorders | - | - | 1.12 | 0.86-1.46 |
|  | Hypertension | - | - | 0.99 | 0.79-1.23 |
|  | Chronic pulmonary disorders | - | - | 1.12 | 0.86-1.46 |
|  | Diabetes | - | - | 1.13 | 0.91-1.40 |
|  | Hypothyroidism | - | - | 1.07 | 0.80-1.43 |
|  | Renal failure | - | - | 0.92 | 0.66-1.28 |
|  | Cancer | - | - | 0.88 | 0.66-1.17 |
|  | Rheumatoid arthritis/collagen vascular diseases | - | - | 0.81 | 0.51-1.29 |
|  | Coagulopathy | - | - | 0.99 | 0.61-1.62 |
|  | Fluid and electrolyte disorders | - | - | 1.14 | 0.75-1.73 |
|  | Depression | - | - | 1.03 | 0.72-1.47 |
|  | Liver disease | - | - | 1.59 | 0.89-2.86 |
| ^1^ comorbidities defined with coding algorithms for defining Elixhauser comorbidities in ICD-9 administrative data (Quan et al. 2005), ≥2 ICD-9 codes per category, hypertension categories merged, diabetes categories merged, different cancer categories merged, drug abuse, alcohol abuse, obesity, weight loss, HIV/AIDS, paralysis, other neurological disorders, blood loss anemia and deficiency anemia not included;  *PIMs with low level of evidence excluded.  ** p<0.05; *** p<0.001 | | | | | |

# eTable 4. Unadjusted and multivariable associations between demographic and clinical factors and the prescribing of potentially inappropriate medications (logistic regression)

| *Demographics and clinical characteristics* | | | | | | *Unadjusted associations* | | *Model 1: Demographic and healthcare utilization variables* | | *Model 2: Model 1 + chronic conditions* | |
| --- | --- | --- | --- | --- | --- | --- | --- | --- | --- | --- | --- |
|  | | | | | | Odds ratio | 95% CI | Odds ratio | 95% CI | Odds ratio | 95% CI |
|  | | | | | | *(N = 17,912)* | | (n = 17,911) | | (n = 17,911) | |
| Age (reference: 65-74) | | | | | | | | | | | |
|  | 75-84 | | | | | 0.85 | 0.69-.1.04 | 0.87 | 0.70-1.07 | 0.88 | 0.71-1.08 |
|  | 85 and above | | | | | 0.66 | 0.51-0.78** | 0.75 | 0.57-0.99** | 0.74 | 0.56-0.98** |
| Male sex (reference: female sex) | | | | | | 1.41 | 1.17-1.70*** | 1.30 | 0.08-1.58** | 1.29 | 1.06-1.58** |
| Hispanic ethnicity (reference: non-hispanic) | | | | | | 1.52 | 9,67-3.45 | 0.99 | 0.35-2.78 | 0.96 | 0.34-2.72 |
| Race (reference: White) | | | | | | | | | | | |
|  | | | | | Asian | 1.27 | 9,52-3.11 | 1.41 | 0.57-3.46 | 1.34 | 0.55-3.32 |
|  | | | | | Black | 1.56 | 1.02-2.36** | 1.55 | 1.02-2.37 | 1.51 | 0.98-2.32 |
|  | | | | | Other | 1.72 | 1.05-2.84** | 1.52 | 0.80-2.89 | 1.49 | 0.78-2.84 |
| Number of inpatient stays (reference: 0) | | | | | | | | | | | |
|  | | | | At least 1 | | 1.39 | 1.11-1.75 | 0.94 | 0.70-1.29 | 0.91 | 0.66-1.25 |
| Number of emergency department visits (reference: 0) | | | | | | | | | | | |
|  | | | | | At least 1 | 1.33 | 1.09-1.62 | 1.10 | 0.85-1.43 | 1.08 | 0.82-1.40 |
| Number of ambulatory visits (reference: ≤9; categories based on quartiles) | | | | | | | | | | | |
|  | | | 10-17 | | | 0.94 | 0.69-1.28 | 0.95 | 0.69-1.29 | 0.95 | 0.69-1.30 |
|  | | | 18-29 | | | 1.39 | 1.05-1.85** | 1.37 | 1.02-1.86** | 1.38 | 1.02-1.88** |
|  | | | ≥30 | | | 2.14 | 1.64-2.79*** | 2.01 | 1.45-2.78*** | 2.02 | 1.44-2.82*** |
| Number of non-acute institutional stays (reference: 0) | | | | | | | | | | | |
|  | | 1 or above | | | | 0.73 | 0.54-0.99** | 0.78 | 0.55-1.10 | 0.74 | 0.52-1.05 |
| Level of polypharmacy (reference: 5-9 medications) | | | | | | | | | | | |
|  | 10 and above | | | | | 1.26 | 0.76-2.10 | 1.15 | 0.68-1.92 | 1.06 | 0.63-1.79 |
| Number of chronic conditions (1-unit increase) | | | | | | 1.05 | 1.01-1.02** | 0.96 | 0.91-1.02 | 0.95 | 0.88-1.02 |
| Number of prescribing orders (1-unit increase) | | | | | | 1.03 | 1.02-1.04*** | 1.03 | 1.02-1.03*** | 1.03 | 1.02-1.03 |
|  | | | | | |  |  |  |  |  | continued |

| *Demographics and clinical characteristics* | | *Unadjusted associations* | | *Model 1: Demographic and healthcare utilization variables* | | *Model 2: Model 1 + chronic conditions* | |
| --- | --- | --- | --- | --- | --- | --- | --- |
|  | | Un-adjusted hazard ratio | 95% CI | Adjusted hazard ratio | 95% CI | Adjusted hazard ratio | 95% CI |
| Types of comorbidities^1^ | | | | | | | |
|  | Congestive heart failure | 1.55 | 1.24-1.94*** | - | - | 1.37 | 1.06-1.78** |
|  | Cardiac arrhythmias | 1.45 | 1.03-2.04** | - | - | 1.02 | 0.70-1.47 |
|  | Valvular disease | 1.08 | 0.81-1.43 | - | - | 0.83 | 0.61-1.12 |
|  | Pulmonary circulation disorders | 1.66 | 0.99-2.75 | - | - | 1.42 | 0.83-2.43 |
|  | Peripheral vascular disorders | 1.14 | 0.88-1.47 | - | - | 1.42 | 0.83-1.43 |
|  | Hypertension | 1.03 | 0.85-1.25 | - | - | 0.98 | 0.78-1.23 |
|  | Chronic pulmonary disorders | 1.22 | 0.96-1.56 | - | - | 1.12 | 0.86-1.46 |
|  | Diabetes | 1.21 | 0.99-1.47 | - | - | 1.16 | 0.93-1.44 |
|  | Hypothyroidism | 0.92 | 0.70-1.22 | - | - | 1.09 | 0.81-1.46 |
|  | Renal failure | 1.12 | 0.82-1.53 | - | - | 0.87 | 0.62-1.22 |
|  | Cancer | 1.12 | 0.86-1.45 | - | - | 0.80 | 0.68-1.20 |
|  | Rheumatoid arthritis/collagen vascular diseases | 0.84 | 0.54-1.33 | - | - | 0.78 | 0.49-1.25 |
|  | Coagulopathy | 1.25 | 0.78-1.99 | - | - | 0.99 | 0.60-1.63 |
|  | Fluid and electrolyte disorders | 1.31 | 0.87-1.97 | - | - | 1.11 | 0.72-1.70 |
|  | Depression | 0.96 | 0.69-1.34 | - | - | 1.00 | 0.70-1.44 |
|  | Liver disease | 1.81 | 1.01-3.26** | - | - | 1.53 | 0.84-2.79 |
| ^1^ comorbidities defined with coding algorithms for defining Elixhauser comorbidities in ICD-9 administrative data (Quan et al. 2005), ≥2 ICD-9 codes per category, hypertension categories merged, diabetes categories merged, different cancer categories merged, drug abuse, alcohol abuse, obesity, weight loss, HIV/AIDS, paralysis, other neurological disorders, blood loss anemia and deficiency anemia not included;  ** p<0.05; *** p<0.001 | | | | | | | |

# eTable 5. Multivariable associations between demographic and clinical factors and the prescribing of potentially inappropriate medications (PIMs) with moderate or high levels of evidence*

| Demographics and clinical characteristics | | | | Model 1: Demographic and healthcare utilization variables | | Model 2: Model 1 + chronic conditions | |
| --- | --- | --- | --- | --- | --- | --- | --- |
|  | | | | Adjusted hazard ratio | 95% CI | Adjusted hazard ratio | 95% CI |
|  | | | | (n = 17,911) | | (n = 17,911) | |
| Age (reference: 65-74) | | | |  | | | |
|  | 75-84 | | | 0.79 | 0.64-0.98** | 0.81 | 0.65-1.01 |
|  | 85 and above | | | 0.70 | 0.52-0.93** | 0.72 | 0.53-0.96** |
| Male sex (reference: female sex) | | | | 1.32 | 1.08-1.61** | 1.34 | 1.09-1.65** |
| Hispanic ethnicity (reference: non-hispanic) | | | | 1.21 | 0.43-3.41 | 1.20 | 0.43-3.39 |
| Race (reference: White) | | | |  | | | |
|  | | | Asian | 1.53 | 0.63-3.69 | 1.43 | 0.59-3.48 |
|  | | | Black | 1.66 | 1.09-2.54** | 1.59 | 1.04-2.44** |
|  | | | Other | 1.47 | 0.76-2.85 | 1.39 | 0.71-2.71 |
| Number of inpatient stays (reference: 0) | | | |  | | | |
|  | | 1 or above | | 0.91 | 0.65-1.26 | 0.93 | 0.66-1.30 |
| Number of emergency department visits (reference: 0) | | | |  | | | |
|  | | | 1 or above | 1.14 | 0.87-1.50 | 1.12 | 0.85-1.48 |
| Number of ambulatory visits (reference: ≤9; categories based on quartiles) | | | | | | | |
|  | 10-17 | | | 0.99 | 0.72-1.36 | 0.99 | 0.73-1.37 |
|  | 18-29 | | | 1.32 | 0.97-1.80 | 1.35 | 0.99-1.85 |
|  | ≥30 | | | 1.91 | 1.36-2.67*** | 1.99 | 1.41-2.81*** |
| Number of non-acute institutional stays (reference: 0) | | | | | | | |
|  | 1 or above | | | 0.89 | 0.63-1.27 | 0.83 | 0.58-1.20 |
| Level of polypharmacy (reference: 5-9 medications) | | | | | | | |
|  | 10 and above | | | 0.82 | 0.44-1.55 | 0.78 | 0.41-1.47 |
| Number of chronic conditions (1-unit increase) | | | | 0.96 | 0.90-1.01 | 0.93 | 0.87-1.00 |
| Number of prescribing orders (1-unit increase) | | | | 1.02 | 1.01-1.02*** | 1.02 | 1.01-1.02*** |
| continued | | | | | | | |

| Demographics and clinical characteristics | | Model 1: Demographic and healthcare utilization variables | | Model 2: Model 1 + chronic conditions | |
| --- | --- | --- | --- | --- | --- |
|  | | Adjusted hazard ratio | 95% CI | Adjusted hazard ratio | 95% CI |
| Types of comorbidities^1^ | |  | | | |
|  | Congestive heart failure | - | - | 1.11 | 0.83-1.48 |
|  | Cardiac arrhythmias | - | - | 0.92 | 0.61-1.40 |
|  | Valvular disease | - | - | 0.76 | 0.54-1.08 |
|  | Pulmonary circulation disorders | - | - | 1.08 | 0.57-2.07 |
|  | Peripheral vascular disorders | - | - | 1.13 | 0.85-1.50 |
|  | Hypertension | - | - | 1.03 | 0.82-1.31 |
|  | Chronic pulmonary disorders | - | - | 1.12 | 0.85-1.49 |
|  | Diabetes | - | - | 1.17 | 0.93-1.46 |
|  | Hypothyroidism | - | - | 1.11 | 0.81-1.52 |
|  | Renal failure | - | - | 1.00 | 0.71-1.41 |
|  | Cancer | - | - | 0.87 | 0.64-1.18 |
|  | Rheumatoid arthritis/collagen vascular diseases | - | - | 0.80 | 0.49-1.32 |
|  | Coagulopathy | - | - | 1.00 | 0.56-1.65 |
|  | Fluid and electrolyte disorders | - | - | 1.24 | 0.81-1.91 |
|  | Depression | - | - | 1.17 | 0.81-1.68 |
|  | Liver disease | - | - | 1.61 | 0.87-2.96 |
| ^1^ comorbidities defined with coding algorithms for defining Elixhauser comorbidities in ICD-9 administrative data (Quan et al. 2005), ≥2 ICD-9 codes per category, hypertension categories merged, diabetes categories merged, different cancer categories merged, drug abuse, alcohol abuse, obesity, weight loss, HIV/AIDS, paralysis, other neurological disorders, blood loss anemia and deficiency anemia not included;  *PIMs with low level of evidence excluded.  ** p<0.05; *** p<0.001 | | | | | |

# eTable 6. Multivariable associations between demographic and clinical factors and the prescribing of potentially inappropriate medications in patients with ≥3 chronic conditions

| Demographics and clinical characteristics | | | | Model 1: Demographic and healthcare utilization variables | | Model 2: Model 1 + chronic conditions | |
| --- | --- | --- | --- | --- | --- | --- | --- |
|  | | | | Adjusted hazard ratio | 95% CI | Adjusted hazard ratio | 95% CI |
|  | | | | (n = 16,134) | | (n = 16,134) | |
| Age (reference: 65-74) | | | |  | | | |
|  | 75-84 | | | 0.89 | 0.71-1.11 | 0.91 | 0.72-1.13 |
|  | 85 and above | | | 0.79 | 0.59-1.05 | 0.78 | 0.58-1.05 |
| Male sex (reference: female sex) | | | | 1.32 | 1.09-1.62** | 1.32 | 1.07-1.62** |
| Hispanic ethnicity (reference: non-hispanic) | | | | 1.16 | 0.41-3.26 | 1.13 | 0.40-3.18 |
| Race (reference: White) | | | |  | | | |
|  | | | Asian | 1.54 | 0.63-3.72 | 1.45 | 0.60-3.53 |
|  | | | Black | 1.57 | 1.02-2.42** | 1.50 | 0.97-2.33 |
|  | | | Other | 1.54 | 0.79-3.00 | 1.50 | 0.77-2.92 |
| Number of inpatient stays (reference: 0) | | | |  | | | |
|  | | 1 or above | | 0.99 | 0.72-1.35 | 0.94 | 0.69-1.30 |
| Number of emergency department visits (reference: 0) | | | | | | | |
|  | | | 1 or above | 1,06 | 0.81-1.39 | 1.03 | 0.79-1.35 |
| Number of ambulatory visits (reference: ≤9; categories based on quartiles) | | | | | | | |
|  | 10-17 | | | 1.03 | 0.73-1.45 | 1.04 | 0.74-1.46 |
|  | 18-29 | | | 1.32 | 0.95-1.84 | 1.35 | 0.96-1.88 |
|  | ≥30 | | | 2.09 | 1.48-2.95*** | 2.12 | 1.48-3.02*** |
| Number of non-acute institutional stays (reference: 0) | | | | | | | |
|  | 1 or above | | | 0.78 | 0.55-1.10 | 0.74 | 0.52-1.05 |
| Level of polypharmacy (reference: 5-9 medications) | | | | | | | |
|  | 10 and above | | | 1.19 | 0.72-1.98 | 1.09 | 0.65-1.81 |
| Number of chronic conditions (1-unit increase) | | | | 0.98 | 0.93-1.04 | 0.96 | 0.89-1.03 |
| Number of prescribing orders (1-unit increase) | | | | 1.02 | 1.01-1.02*** | 1.02 | 1.01-1.02*** |
| continued | | | | | | | |

| Demographics and clinical characteristics | | Model 1: Demographic and healthcare utilization variables | | Model 2: Model 1 + chronic conditions | |
| --- | --- | --- | --- | --- | --- |
|  | | Adjusted hazard ratio | 95% CI | Adjusted hazard ratio | 95% CI |
| Types of comorbidities^1^ | |  | | | |
|  | Congestive heart failure | - | - | 1.43 | 1.10-1.86** |
|  | Cardiac arrhythmias | - | - | 1.01 | 0.70-1.46 |
|  | Valvular disease | - | - | 0.86 | 0.63-1.16 |
|  | Pulmonary circulation disorders | - | - | 1.41 | 0.83-2.38 |
|  | Peripheral vascular disorders | - | - | 1.10 | 0.84-1.44 |
|  | Hypertension | - | - | 1.02 | 0.81-1.29 |
|  | Chronic pulmonary disorders | - | - | 1.14 | 0.88-1.49 |
|  | Diabetes | - | - | 1.22 | 0.98-1.51 |
|  | Hypothyroidism | - | - | 1.11 | 0.82-1.48 |
|  | Renal failure | - | - | 0.91 | 0.65-1.27 |
|  | Cancer | - | - | 0.88 | 0.66-1.18 |
|  | Rheumatoid arthritis/collagen vascular diseases | - | - | 0.86 | 0.54-1.36 |
|  | Coagulopathy | - | - | 0.94 | 0.57-1.54 |
|  | Fluid and electrolyte disorders | - | - | 0.94 | 0.61-1.48 |
|  | Depression | - | - | 1.05 | 0.73-1.50 |
|  | Liver disease | - | - | 1.57 | 0.88-2.83 |
| ^1^ comorbidities defined with coding algorithms for defining Elixhauser comorbidities in ICD-9 administrative data (Quan et al. 2005), ≥2 ICD-9 codes per category, hypertension categories merged, diabetes categories merged, different cancer categories merged, drug abuse, alcohol abuse, obesity, weight loss, HIV/AIDS, paralysis, other neurological disorders, blood loss anemia and deficiency anemia not included;  ** p<0.05; *** p<0.001 | | | | | |

# eTable 7. Multivariable associations between demographic and clinical factors and the prescribing of potentially inappropriate medications in patients with min. 2 ambulatory visits recorded in the electronic medical records during the baseline period

| Demographics and clinical characteristics | | | | Model 1: Demographic and healthcare utilization variables | | Model 2: Model 1 + chronic conditions | |
| --- | --- | --- | --- | --- | --- | --- | --- |
|  | | | | Adjusted hazard ratio | 95% CI | Adjusted hazard ratio | 95% CI |
|  | | | | (n = 17,466) | | (n = 17,466) | |
| Age (reference: 65-74) | | | |  | | | |
|  | 75-84 | | | 0.87 | 0.70-1.07 | 0.88 | 0.71-1.09 |
|  | 85 and above | | | 0.77 | 0.58-1.01 | 0.76 | 0.57-1.01 |
| Male sex (reference: female sex) | | | | 1.24 | 1.03-1.50** | 1.23 | 1.01-1.50** |
| Hispanic ethnicity (reference: non-hispanic) | | | | 0.93 | 0.34-2.51 | 0.92 | 0.34-2.49 |
| Race (reference: White) | | | |  | | | |
|  | | | Asian | 1.43 | 0.59-3.46 | 1.36 | 0.56-3.29 |
|  | | | Black | 1.47 | 0.95-2.26 | 1.42 | 0.92-2.19 |
|  | | | Other | 1.73 | 0.95-3.15 | 1.67 | 0.91-3.06 |
| Number of inpatient stays (reference: 0) | | | |  | | | |
|  | | 1 or above | | 0.98 | 0.73-1.33 | 0.94 | 0.69-1.29 |
| Number of emergency department visits (reference: 0) | | | |  | | | |
|  | | | 1 or above | 1.13 | 0.69-1.46 | 1.10 | 0.85-1.43 |
| Number of ambulatory visits (reference: ≤9; categories based on quartiles) | | | | | | | |
|  | 10-17 | | | 1.04 | 0.75-1.43 | 1.04 | 0.75-1.43 |
|  | 18-29 | | | 1.53 | 1.12-2.08** | 1.53 | 1.12-2.10** |
|  | ≥30 | | | 2.29 | 1.64-3.19*** | 2.27 | 1.62-3.20*** |
| Number of non-acute institutional stays (reference: 0) | | | | | | | |
|  | 1 or above | | | 0.78 | 0.55-1.11 | 0.73 | 0.52-1.05 |
| Level of polypharmacy (reference: 5-9 medications) | | | | | | | |
|  | 10 and above | | | 1.18 | 0.71-1.95 | 1.08 | 0.65-1.79 |
| Number of chronic conditions (1-unit increase) | | | | 0.96 | 0.91-1.01 | 0.95 | 0.88-1.01 |
| Number of prescribing orders (1-unit increase) | | | | 1.02 | 1.02-1.02*** | 1.02 | 1.01-1.02*** |
| continued | | | | | | | |

| Demographics and clinical characteristics | | Model 1: Demographic and healthcare utilization variables | | Model 2: Model 1 + chronic conditions | |
| --- | --- | --- | --- | --- | --- |
|  | | Adjusted hazard ratio | 95% CI | Adjusted hazard ratio | 95% CI |
| Types of comorbidities^1^ | |  | | | |
|  | Congestive heart failure | - | - | 1.40 | 1.09-1.81** |
|  | Cardiac arrhythmias | - | - | 1.03 | 0.71-1.47 |
|  | Valvular disease | - | - | 0.85 | 0.63-1.15 |
|  | Pulmonary circulation disorders | - | - | 1.41 | 0.84-2.38 |
|  | Peripheral vascular disorders | - | - | 1.10 | 0.84-1.43 |
|  | Hypertension | - | - | 0.96 | 0.77-1.20 |
|  | Chronic pulmonary disorders | - | - | 1.10 | 0.84-1.43 |
|  | Diabetes | - | - | 1.18 | 0.95-1.47 |
|  | Hypothyroidism | - | - | 1.07 | 0.80-1.44 |
|  | Renal failure | - | - | 0.91 | 0.65-1.26 |
|  | Cancer | - | - | 0.88 | 0.66-1.17 |
|  | Rheumatoid arthritis/collagen vascular diseases | - | - | 0.81 | 0.51-1.28 |
|  | Coagulopathy | - | - | 0.95 | 9,58-1.56 |
|  | Fluid and electrolyte disorders | - | - | 1.10 | 0.73-1.67 |
|  | Depression | - | - | 1.06 | 0.74-1.52 |
|  | Liver disease | - | - | 1.55 | 0.86-2.77 |
| ^1^ comorbidities defined with coding algorithms for defining Elixhauser comorbidities in ICD-9 administrative data (Quan et al. 2005), ≥2 ICD-9 codes per category, hypertension categories merged, diabetes categories merged, different cancer categories merged, drug abuse, alcohol abuse, obesity, weight loss, HIV/AIDS, paralysis, other neurological disorders, blood loss anemia and deficiency anemia not included;  ** p<0.05; *** p<0.001 | | | | | |

# eTable 8. Multivariable associations between demographic and clinical factors and the prescribing of potentially inappropriate medications in patients: Sensitivity analysis with claims-based frailty index

| **Demographics and clinical characteristics** | | | | **Full model plus frailty index^1^** | |
| --- | --- | --- | --- | --- | --- |
|  | | | | Adjusted hazard ratio | 95% CI |
|  | | | | (n = 17,911) | |
| Age (reference: 65-74) | | | |  | |
|  | 75-84 | | | 0.89 | 0.73-1.10 |
|  | 85 and above | | | 0.78 | 0.58-1.02 |
| Male sex (reference: female sex) | | | | 1.31 | 1.08-1.59** |
| Hispanic ethnicity (reference: non-hispanic) | | | | 0.94 | 0.34-2.54 |
| Race (reference: White) | | | |  | |
|  | | | Asian | 1.28 | 0.53-3.11 |
|  | | | Black | 1.47 | 0.97-2.24 |
|  | | | Other | 1.58 | 0.86-2.89 |
| Number of inpatient stays (reference: 0) | | | |  | |
|  | | 1 or above | | 1.03 | 0.75-1.42 |
| Number of emergency department visits (reference: 0) | | | |  | |
|  | | | 1 or above | 1.19 | 0.91-1.55 |
| Number of ambulatory visits (reference: ≤9; categories based on quartiles) | | | |  | |
|  | 10-17 | | | 0.99 | 0.72-1.34 |
|  | 18-29 | | | 1.48 | 1.09-1.99** |
|  | ≥30 | | | 2.22 | 1.60-3.09*** |
| Number of non-acute institutional stays (reference: 0) | | | |  | |
|  | 1 or above | | | 0.93 | 0.64-1.34 |
| Level of chronic polypharmacy (reference: 5-9 medications) | | | |  | |
|  | 10 and above | | | 1.11 | 0.66-1.84 |
| Number of chronic conditions (1-unit increase) | | | | 0.98 | 0.91-1.05 |
| Number of prescribing orders (1-unit increase) | | | | 1.02 | 1.01-1.02*** |
|  | | | | continued | |

| **Demographics and clinical characteristics** | | **Full model plus frailty index^1^** | |
| --- | --- | --- | --- |
|  | | 95% CI | 95% CI |
| Types of comorbidities^2^ | |  | |
|  | Congestive heart failure | 1.44 | 1.11-1.86** |
|  | Cardiac arrhythmias | 1.03 | 0.72-1.48 |
|  | Valvular disease | 0.86 | 0.64-1.16 |
|  | Pulmonary circulation disorders | 1.39 | 0.82-2.33 |
|  | Peripheral vascular disorders | 1.12 | 0.86-1.46 |
|  | Hypertension | 1.00 | 0.80-1.24 |
|  | Chronic pulmonary disorders | 1.18 | 0.91-1.54 |
|  | Diabetes | 1.21 | 0.98-1.50 |
|  | Hypothyroidism | 1.05 | 0.78-1.40 |
|  | Renal failure | 0.96 | 0.69-1.33 |
|  | Cancer | 0.84 | 0.64-1.12 |
|  | Rheumatoid arthritis/collagen vascular diseases | 0.80 | 0.51-1.27 |
|  | Coagulopathy | 0.96 | 0.59-1.57 |
|  | Fluid and electrolyte disorders | 1.11 | 0.73-1.68 |
|  | Depression | 1.13 | 0.791.62 |
|  | Liver disease | 1.47 | 0.82-2.64 |
| Frailty index (per unit increase) | | 0.08 | 0.01-0.48** |
| ^1^Kim DH, Schneeweiss S, Lipsitz LA, Glynn R, Rockwood K, Avorn J. Measuring Frailty in Medicare Data: Development and Validation of a Claims-Based Frailty Index. J Gerontol A Biol Sci Med Sci. 2018; 73: 980-987. doi: 10.1093/gerona/glx229. PMID: 29244057; PMCID: PMC6001883; ^2^comorbidities defined with coding algorithms for defining Elixhauser comorbidities in ICD-9 administrative data (Quan et al. 2005), ≥2 ICD-9 codes per category, hypertension categories merged, diabetes categories merged, different cancer categories merged, drug abuse, alcohol abuse, obesity, weight loss, HIV/AIDS, paralysis, other neurological disorders, blood loss anemia and deficiency anemia not included; ** p<0.05; *** p<0.001 | | | |
